# Additive value of [^18^F]PI-2620 perfusion imaging in progressive supranuclear palsy and corticobasal syndrome

**DOI:** 10.1007/s00259-022-05964-w

**Published:** 2022-09-14

**Authors:** Sabrina Katzdobler, Alexander Nitschmann, Henryk Barthel, Gerard Bischof, Leonie Beyer, Ken Marek, Mengmeng Song, Olivia Wagemann, Carla Palleis, Endy Weidinger, Anne Nack, Urban Fietzek, Carolin Kurz, Jan Häckert, Theresa Stapf, Christian Ferschmann, Maximilian Scheifele, Florian Eckenweber, Gloria Biechele, Nicolai Franzmeier, Anna Dewenter, Sonja Schönecker, Dorothee Saur, Matthias L. Schroeter, Jost-Julian Rumpf, Michael Rullmann, Andreas Schildan, Marianne Patt, Andrew W. Stephens, Thilo van Eimeren, Bernd Neumaier, Alexander Drzezga, Adrian Danek, Joseph Classen, Katharina Bürger, Daniel Janowitz, Boris-Stephan Rauchmann, Sophia Stöcklein, Robert Perneczky, Florian Schöberl, Andreas Zwergal, Günter U. Höglinger, Peter Bartenstein, Victor Villemagne, John Seibyl, Osama Sabri, Johannes Levin, Matthias Brendel, for the German Imaging Initiative for Tauopathies (GII4T)

**Affiliations:** 1 Department of Neurology, University Hospital of Munich, LMU Munich, Munich, Germany; 2 German Center for Neurodegenerative Diseases (DZNE), Munich, Germany; 3 Munich Cluster for Systems Neurology (SyNergy), Munich, Germany; 4 Department of Nuclear Medicine, University Hospital of Munich, LMU Munich, Marchioninistraße 15, 81377 Munich, Germany; 5 Department of Nuclear Medicine, University Hospital of Leipzig, Leipzig, Germany; 6 Department of Nuclear Medicine, University Hospital Cologne, Cologne, Germany; 7 Molecular Organization of the Brain, Institute for Neuroscience and Medicine, INM-2), Jülich, Germany; 8 InviCRO, LLC, Boston, MA USA; 9 Molecular Neuroimaging, A Division of inviCRO, New Haven, CT USA; 10 Department of Psychiatry and Psychotherapy, University Hospital, LMU Munich, Munich, Germany; 11 Department of Psychiatry, Psychotherapy and Psychosomatics, Medical Faculty, University of Augsburg, BKH Augsburg, Augsburg, Germany; 12 Institute for Stroke and Dementia Research, University Hospital of Munich, LMU Munich, Munich, Germany; 13 Department of Neurology, University of Leipzig Medical Center, Leipzig, Germany; 14 Clinic for Cognitive Neurology, University of Leipzig, Leipzig, Germany; 15 LIFE - Leipzig Research Center for Civilization Diseases, University of Leipzig, Leipzig, Germany; 16 Max Planck Institute for Human Cognitive and Brain Sciences, Leipzig, Germany; 17 Life Molecular Imaging GmbH, Berlin, Germany; 18 Institute for Neuroscience and Medicine (INM-3), Cognitive Neuroscience, Research Centre Juelich, Juelich, Germany; 19 German Center for Neurodegenerative Diseases (DZNE), Bonn, Germany; 20 Department of Radiology, University Hospital of Munich, LMU Munich, Munich, Germany; 21 Ageing Epidemiology Research Unit (AGE), School of Public Health, Imperial College, London, UK; 22 Department of Neurology, Hannover Medical School, Hannover, Germany; 23 Department of Molecular Imaging & Therapy, Austin Health, Heidelberg, VIC Australia; 24 Department of Medicine, Austin Health, The University of Melbourne, Melbourne, VIC Australia; 25 Department of Psychiatry, University of Pittsburgh, Pittsburgh, PA USA

**Keywords:** Tau, PET, [^18^F]PI-2620, Perfusion, Neuronal injury

## Abstract

**Purpose:**

Early after [^18^F]PI-2620 PET tracer administration, perfusion imaging has potential for regional assessment of neuronal injury in neurodegenerative diseases. This is while standard late-phase [^18^F]PI-2620 tau-PET is able to discriminate the 4-repeat tauopathies progressive supranuclear palsy and corticobasal syndrome (4RTs) from disease controls and healthy controls. Here, we investigated whether early-phase [^18^F]PI-2620 PET has an additive value for biomarker based evaluation of 4RTs.

**Methods:**

Seventy-eight patients with 4RTs (71 ± 7 years, 39 female), 79 patients with other neurodegenerative diseases (67 ± 12 years, 35 female) and twelve age-matched controls (69 ± 8 years, 8 female) underwent dynamic (0–60 min) [^18^F]PI-2620 PET imaging. Regional perfusion (0.5–2.5 min p.i.) and tau load (20–40 min p.i.) were measured in 246 predefined brain regions [standardized-uptake-value ratios (SUVr), cerebellar reference]. Regional SUVr were compared between 4RTs and controls by an ANOVA including false-discovery-rate (FDR, *p* < 0.01) correction. Hypoperfusion in resulting 4RT target regions was evaluated at the patient level in all patients (mean value − 2SD threshold). Additionally, perfusion and tau pattern expression levels were explored regarding their potential discriminatory value of 4RTs against other neurodegenerative disorders, including validation in an independent external dataset (*n* = 37), and correlated with clinical severity in 4RTs (PSP rating scale, MoCA, activities of daily living).

**Results:**

Patients with 4RTs had significant hypoperfusion in 21/246 brain regions, most dominant in thalamus, caudate nucleus, and anterior cingulate cortex, fitting to the topology of the 4RT disease spectrum. However, single region hypoperfusion was not specific regarding the discrimination of patients with 4RTs against patients with other neurodegenerative diseases. In contrast, perfusion pattern expression showed promise for discrimination of patients with 4RTs from other neurodegenerative diseases (AUC: 0.850). Discrimination by the combined perfusion-tau pattern expression (AUC: 0.903) exceeded that of the sole tau pattern expression (AUC: 0.864) and the discriminatory power of the combined perfusion-tau pattern expression was replicated in the external dataset (AUC: 0.917). Perfusion but not tau pattern expression was associated with PSP rating scale (*R* = 0.402; *p* = 0.0012) and activities of daily living (*R* =  − 0.431; *p* = 0.0005).

**Conclusion:**

[^18^F]PI-2620 perfusion imaging mirrors known topology of regional hypoperfusion in 4RTs. Single region hypoperfusion is not specific for 4RTs, but perfusion pattern expression may provide an additive value for the discrimination of 4RTs from other neurodegenerative diseases and correlates closer with clinical severity than tau pattern expression.

**Supplementary Information:**

The online version contains supplementary material available at 10.1007/s00259-022-05964-w.

## Introduction

The identification of specific biomarkers that allow for early detection of tau pathology in four-repeat tauopathies (4RTs) will become crucial for target engagement in tau targeting treatment trials. In this regard, imaging with the next-generation tau-PET tracer [^18^F]PI-2620 facilitated discrimination of patients with a clinical diagnosis of the 4RTs progressive supranuclear palsy (PSP) [1] and corticobasal syndrome (CBS) [2] from healthy controls, non-tauopathy Parkinson syndromes and Alzheimer’s disease (AD). [^18^F]PM-PBB3 also has the potential to differentiate 4RTs in vivo [3]. Neurodegeneration of cortical and subcortical brain regions is a common feature of 4RTs [4, 5], comprising a relevant objective parameter of disease progression [6] in AD as the most frequent tauopathy [7]. For AD, it has been proposed to classify the disease according to biomarkers for amyloid, tau, and neurodegeneration by the A/T/N scheme [8]. In this classification scheme, neurodegeneration on a biomarker level can be determined in vivo by different diagnostic approaches: (i) atrophy in structural magnetic resonance imaging (MRI), (ii) levels of total tau in cerebrospinal fluid, (iii) hypometabolism in [^18^F]fluorodeoxyglucose-(FDG)-PET, (iv) or hypoperfusion with several imaging techniques such as single-photon-emission-computed-tomography (SPECT). Brain atrophy in MRI as well as region-specific hypometabolism patterns in FDG-PET are well established in the diagnostic work-up in 4RTs [9]. However, an equivalent concept to A/T/N of combining simultaneous visualization of tau pathology and neurodegeneration in 4RTs has not yet been established. Recently, we reported that early-phase imaging of β-amyloid PET closely matches the pattern of glucose uptake in CBS [10] and we found that early-phase imaging of [^18^F]PI-2620 tau-PET could also serve as a surrogate of brain perfusion in mixed neurodegenerative disorders [11]. With respect to cost, radiation exposure, and patient burden, such “one-stop shop” protocols provide the opportunity to examine two important diagnostic and potentially also prognostic biomarkers simultaneously with one procedure. We hypothesized that early-phase imaging with [^18^F]PI-2620 PET mirrors the known neurodegeneration pattern in the brain of 4RT patients when compared to controls. Furthermore, we hypothesized that 4RT-related perfusion expression patterns may facilitate the discrimination of 4RT patients against other neurodegenerative diseases. Since the utility of imaging biomarkers for diagnosis and disease progression may well differ [12], we directly correlated perfusion patterns as well as the amount and pattern of tau pathology with clinical and functional scores in 4RTs.

## Material and methods

### Patient enrolment and study design

Seventy-eight patients with possible or probable 4RTs (71 ± 7 years, 39 female) were enrolled at the departments of neurology and psychiatry of the LMU Munich. Tau-PET data of PSP [1] and CBS [2] patients were previously published elsewhere. The 4RT cohort consisted of 30 patients with PSP Richardson syndrome (PSP-RS), 23 cases with predominant corticobasal syndrome (PSP-CBS), five cases with predominant Parkinsonism (PSP-P), two cases with predominant frontal cognitive dysfunction (PSP-F), and one case each with predominant speech/language disorder (PSP-SL), with primary lateral sclerosis (PSP-PLS) and with pure akinesia with gait freezing (PSP-PGF), and 15 patients fulfilled possible CBS criteria. Clinical diagnoses were based on MDS PSP and Armstrong criteria [9, 13]. All included patients fulfilling CBS criteria had a negative amyloid-PET scan or negative Aβ in CSF (Aβ_42_ and Aβ_42_/Aβ_40_ ratio) to rule out AD pathophysiology [2]. Seventy-nine patients with suspected neurodegenerative diseases other than 4RT movement disorders (PSP, CBS) were enrolled in the same time period (Oct 2018–Apr 2021) at LMU Munich and used as a comparative dataset. This cohort underwent an equal clinical workup and it was composed of patients belonging to the AD continuum (*n* = 47; all amyloid-PET positive or positive Aβ in CSF (Aβ_42_ and Aβ_42_/Aβ_40_ ratio)), α-synucleinopathies (*n* = 12), FTD (*n* = 10), and other neurodegenerative diseases (i.e. anti-IgLON5 syndrome or Down syndrome; *n* = 10). Twelve amyloid-negative healthy controls without cognitive decline or motor disability that matched in age and sex were used from published datasets [1, 2] and additional recruitment in Munich. Furthermore, an independent dataset composed of 21 patients with 4RTs and 16 patients with other neurodegenerative disorders was used from centers in Leipzig, Cologne, and New Haven [1]. An overview on all used data is provided in Table 1.

**Table 1:** Demographics and clinical scores of the study cohorts

Study group	4RTs LMU	Other neurodegenerative diseases LMU	4RTs external validation dataset	Other neurodegenerative diseases external validation dataset	Healthy controls
*n*	78	79	21	16	12
Subgroups	30 PSP-RS 23 PSP-CBS 2 PSP-F 5 PSP-P 1 PSP-SL 1 PSP-PLS 1 PSP-PGF 15 CBS	47 AD 10 FTLD 12 aSyn 10 other	17 PSP-RS 2 PSP-F 2 PSP-P	9 AD 5 FTLD 2 other	n.a
Age (years)	71.2 ± 7.1	66.6 ± 11.6	70.7 ± 5.8	67.7 ± 10.1	68.5 ± 7.5
Sex	39 female / 39 male	35 female / 44 male	8 female / 13 male	7 female / 9 male	8 female / 4 male
PSP Rating Scale	30.0 ± 12.6	22.9 ± 7.3	39.8 ± 15.2	n.a	n.a
Possible (*n*)/probable (*n*) 4RT	30/48	n.a	3/18	n.a	n.a
Disease duration (months)	38.0 ± 28.6	32.0 ± 26.0	49.4 ± 29.3	36.9 ± 22.0	n.a
MoCA	21.9 ± 5.5	20.3 ± 6.6	n.a	14.7 ± 8.1	28.9 ± 1.2
SEADL	60.5 ± 19.3	68.7 ± 13.0	52.0 ± 24.6	n.a	n.a

*4RTs*, 4-repeat tauopathies; *PSP*, progressive supranuclear palsy; *RS*, Richardson syndrome; *CBS*, corticobasal syndrome; *F*, frontal; *P*, Parkinson; *SL*, speech/language; *PLS*, primary lateral sclerosis; *PGF*, pure akinesia with gait freezing; *aSyn*, α-synucleinopathies; *AD*, Alzheimer’s disease; *FTLD*, fronto-temporal lobe dementia; *MoCA*, Montreal Cognitive Assessment; *SEADL*, Schwab and England activities of daily living; *n.a*., not available

Regional PET quantification of perfusion imaging was compared between 4RTs and healthy controls. Target region positivity was evaluated at the individual patient level in 4RTs and in the comparative dataset of other neurodegenerative disorders. Additionally, perfusion and tau pattern expression levels were used for discrimination of 4RTs and other neurodegenerative disorders, including validation in the independent external dataset.

All patients and controls provided informed written consent. The study was conducted in accordance with the principles of the Declaration of Helsinki, and approval for scientific data analysis was obtained from the local ethics committee (application numbers 17–569, 19–022).

### PET imaging

#### Radiosynthesis

Radiosynthesis of [^18^F]PI-2620 was achieved by nucleophilic substitution on a BOC-protected nitro precursor using an automated synthesis module (Synthera, IBA Louvain-la-neuve, Belgium). The protecting group was cleaved under the radiolabelling conditions. The product was purified by semipreparative HPLC. Radiochemical purity was ≥ 97%. Non-decay corrected yields were about 30% with a molar activity of 3∙10^6^ GBq/mmol at the end of synthesis.

#### PET acquisition and preparation

The main cohort of this study was scanned at the Department of Nuclear Medicine, LMU Munich, with a Biograph 64 or a Siemens mCT PET/CT scanner (both Siemens, Erlangen, Germany). A low-dose CT scan preceded the PET acquisition and served for attenuation correction. [^18^F]PI-2620-PET was performed in a full dynamic 0–60 min setting initiated upon intravenous injection (~ 10 s) of 185 ± 10 MBq of the tracer. Dynamic emission recordings were framed into 6 × 30 s, 4 × 60 s, 4 × 120 s, and 9 × 300 s. PET data were reconstructed iteratively (4 iterations, 21 subsets, 5.0 mm Gauss/5 iterations, 24 subsets, 5.0 mm Gauss) with a matrix size of 336 × 336 × 109/ 400 × 400 × 148, a voxel size of 1.018 × 1.018 × 2.027/ 1.018 × 1.018 × 1.500 mm^3^/and a slice thickness of 2.027/1.500 mm. Standard corrections with regard to scatter, decay, and random counts were used. Data from Hofmann phantoms were used to obtain scanner-specific filter functions which were then consequently used to generate images with a similar resolution (FWHM: 9 × 9 × 10 mm), following the ADNI image harmonization procedure [14].

Controls and the external validation dataset were scanned at different imaging units (Leipzig: Siemens Biograph mMR, Siemens, Erlangen, Germany; New Haven: Siemens ECAT EXACT HR + , Siemens, Erlangen, Germany; Melbourne: Philips Gemini TF 64 PET/CT, Eindhoven, The Netherlands; Cologne: Siemens mCT PET/CT, Siemens, Erlangen, Germany) using the same established scanning protocol. Details on all scanners, as well as acquisition and reconstruction parameter, are provided in the Supplement of our previous study [1].

#### Image processing

All image data were processed and analysed using PMOD (version 3.9, PMOD Technologies Ltd., Zurich, Switzerland). For spatial normalization, tracer-specific templates in the MNI space were created for early-phase (0.5–2.5 min [11]) and late-phase (20–40 min [15]) [^18^F]PI-2620 data as described previously [16]. Based upon our previous work [1], we created optimized templates by use of 35 randomly selected individuals with a structural high-resolution 3D T1-weighted image (MPRAGE). Early-phase and late-phase [^18^F]PI-2620 images were spatially normalized to MNI space by applying a non-linear transformation (brain normalization settings: nonlinear warping, 8 mm input smoothing, equal modality, 16 iterations, frequency cutoff 3, regularization 1.0, no thresholding). The cerebellum (excluding the dentate nucleus and superior layers) was used as a reference region for scaling of early- and late-phase [^18^F]PI-2620 images. Standardised uptake value ratios (SUVr) of all 246 regions of interest of the Brainnetome atlas [17] were extracted and used for data analysis. A subset of *n* = 42 patients with 4RTs and *n* = 8 healthy controls was processed including partial volume effect correction (PVEC) and the perfusion pattern differences of 4RTs and healthy controls were compared between uncorrected and PVE-corrected data. For partial volume effect correction (PVEC), we used the Gaussian Transfer Method (GTM) [18] as implemented in the PETPVC toolbox, i.e. a pre-established software package designed for partial volume correction of PET data. We specifically chose the GTM approach, due to its suitability for partial volume correction of region of interest-based data [18]. The exact mathematical approach of the GTM as implemented in the PETPVC toolbox has been described in detail previously [19] and can be accessed online (https://github.com/UCL/PETPVC). For PVEC correction, we used data of participants with both 3 T T1w MRI and PI-2620 tau-PET data available. T1w MRI images were warped to MNI space using the non-linear high-dimensional warping that is implemented in the Advanced Normalization Tools (ANTs) package (http://stnava.github.io/ANTs/). PET images were rigidly co-registered to the native-space T1w images using ANTs, and the Brainnetome atlas was subsequently warped from MNI to PET space by combining the reversed non-linear warping (i.e. T1w to MNI) and linear registration parameters (i.e. PET to T1w). In native PET space, we then used the PETPVC toolbox2 and applied the GTM algorithm using the scanners Point Spread Function, in order to determine partial volume effect corrected PET data for each ROI of the Brainnetome atlas. Pons scaling was used for the PVEC subset in order to minimize methodological induced effects on the reference tissue.

#### Statistical analysis

All statistical analyses were performed using SPSS (version 26.0, IBM, Armonk, NY, USA).

*Hypoperfusion pattern:* Regional early-phase SUVr of all 246 Brainnetome regions were compared between 4RTs and healthy controls by an ANOVA including false-discovery-rate (FDR, *p* < 0.01) correction for multiple comparisons as well as adjustment for age and sex.

*Single region classifier:* The resulting 4RT target regions were subject to an individual subject classifier. Regional SUVr ≤ mean value (MV) − 2 standard deviations (SD) of the healthy controls were defined as significant regional hypoperfusion. Here, one affected target region defined the subject as positive (dichotomous) for a 4RT-like hypoperfusion scan. This classification was performed in all patients with 4RTs and other not neurodegenerative diseases, scanned in Munich. Sensitivity, specificity, and positive and negative predictive values were calculated for identification of patients with 4RTs in this cohort. This procedure was repeated for the presence of three and five affected target regions and with MV-2.5 SD and MV-3.0 SD thresholds in order to validate the results against altered sensitivity.

*Discrimination by pattern expression:* A principal component analysis (PCA) was performed to test pattern expression levels of perfusion and tau signal for discrimination of 4RTs from other neurodegenerative disorders [20]. The PCA was performed separately for early- and late-phase of [^18^F]PI-2620 imaging. Prior to the PCA, the linear relationship of the data was tested by a correlation matrix and items with a correlation coefficient < 0.3 were discarded. The Kaiser–Meyer–Olkin (KMO) measure and Bartlett’s test of sphericity were used to test for sampling adequacy and suitability for data reduction. Components with an Eigenvalue > 1.0 were extracted and a varimax rotation was selected. Resulting principal components were subject to a regression analysis to calculate their estimation value for 4RTs. Weighting factors obtained from the regression were then multiplied with each principal component to compute a single 4RT-related pattern expression score (4RTRP) per patient [20]. Validation in the independent external dataset was performed by applying the obtained weighting factors to the PCs of single cases. The 4RTRP expression scores were subject to a receiver operating characteristics (ROC) analysis to explore their potential for discrimination of 4RTs from other neurodegenerative disorders. Resulting area under the curve (AUC) values were compared for discriminatory performance by perfusion 4RTRP expression, tau 4RTRP expression, and the summation of both expression scores.

*Correlation with clinical severity:* Perfusion 4RTRP expression, tau 4RTRP expression, and the summation of both expression scores of patients with 4RTs were correlated with the clinical severity scores PSP rating scale, Montreal cognitive assessment (MoCA), and Schwab and England activities of daily living (SEADL) using a partial regression corrected for disease duration (time between symptom onset and PET), age and sex.

## Results

### Demographics

A total of 157 patients with neurodegenerative disorders (age: 69.0 ± 9.9 years, 74 female) and twelve healthy controls (age: 68.5 ± 7.5 years, 8 female) were included in the main analysis. This sample consisted of 78 patients with suspected, possible, or probable 4RTs (age: 71.2 ± 7.1 years, 39 female) and 79 patients with other neurodegenerative diseases (59% AD, 13% FTD, 15% aSyn, 13% other; age: 66.6 ± 11.6 years, 35 female). The validation cohort consisted of 21 patients with suspected, possible or probable 4RTs (age: 70.7 ± 5.8 years, 8 female) and 16 patients with other neurodegenerative disorders (age: 67.7 ± 10.1 years, 7 female). For details of the study population, see Table 1. The clinical follow-up time of the cohort of patients with 4RTs and mixed neurodegenerative diseases was 18 ± 10 months with 71% of patients returning for follow-up visits with patients continuously fulfilling diagnostic criteria without change in diagnosis at their follow-up visits.

### *Early-phase [*^*18*^*F]PI-2620 PET imaging in 4RTs resembles topology of neuronal injury*

**Fig. 1:**
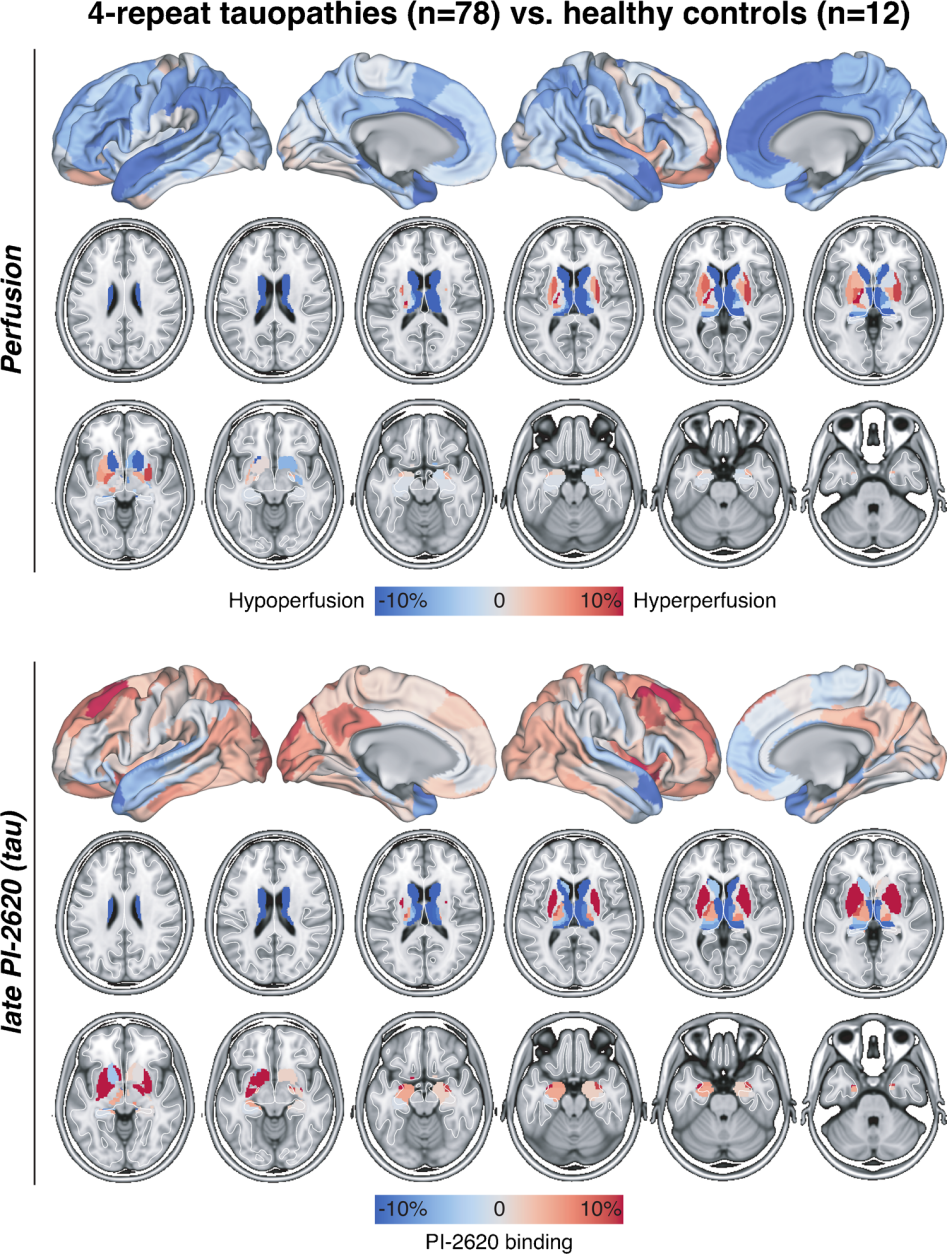
Regional pattern of perfusion alterations in 4RTs when compared to healthy controls. Top: Percentage differences of perfusion imaging are illustrated without significance thresholding for cortical (surface projections) and subcortical (axial slices) Brainnetome regions. Bottom: Percentage differences of late-phase [18F]PI-2620 are illustrated for comparison purpose and reflected previously published data [1, 2]

When compared to healthy controls, significant (FDR, *p* < 0.01) regional hypoperfusion in the 4RT cohort was observed in the thalamus, caudate nucleus, anterior cingulate cortex, and cortical regions of the frontal (superior and inferior frontal gyri) and the temporal lobe (superior gyrus and mesial temporal lobe), comprising 21 out of 246 brain regions of the Brainnetome atlas (Table 2, Fig. 1). Visually discernible hyperperfused regions (i.e. putamen) did not survive FDR correction. There was no hemispheric dominance of hypoperfusion (11 right/10 left). PVEC in a subset of patients with 4RT and controls revealed a similar hypoperfusion pattern when compared to uncorrected data (Supplemental Fig. 1).

**Table 2:** Regions with significant hypoperfusion in 4RTs when compared to healthy controls

Brainnetome region	Compartment	*p*-value (FDR, *p* < 0.01)	%-difference to HC
dCa_L	Basal ganglia	0.0024	− 28.34%
dCa_R	Basal ganglia	0.0098	− 27.75%
cTtha_R	Thalamus	0.0059	− 27.73%
rTtha_R	Thalamus	4.07E-06	− 27.48%
rTtha_L	Thalamus	0.0003	− 21.08%
mPFtha_R	Thalamus	0.0003	− 19.81%
vCa_R	Basal ganglia	0.0058	− 14.69%
mPMtha_L	Thalamus	0.0017	− 14.64%
mPFtha_L	Thalamus	0.0026	− 14.36%
A38l_L	Temporal lobe	0.0018	− 14.23%
A32sg_R	Limbic	0.0030	− 14.15%
A24cd_R	Limbic	0.0099	− 13.92%
A32p_R	Limbic	0.0035	− 13.16%
A9m_R	Frontal lobe	0.0032	− 12.78%
A10m_R	Frontal lobe	0.0032	− 12.45%
A38l_R	Temporal lobe	0.0021	− 11.38%
A44d_L	Frontal lobe	0.0018	− 10.99%
A38m_L	Temporal lobe	0.0054	− 10.34%
A39rd_L	Parietal lobe	0.0037	− 9.62%
TH_L	Temporal lobe	0.0057	− 9.45%
IFS_L	Frontal lobe	0.0098	− 8.96%

All regions reaching a significance threshold of *p* < 0.01 after false discovery rate (FDR) correction for multiple comparisons are listed together with their *p*-values and %-difference compared to healthy controls (HCs). First column provides official Brainnetome region label

### Regional hypoperfusion in individual brain regions of 4RTs is not specific in a cohort of patients with mixed neurodegenerative disorders

**Fig. 2:**
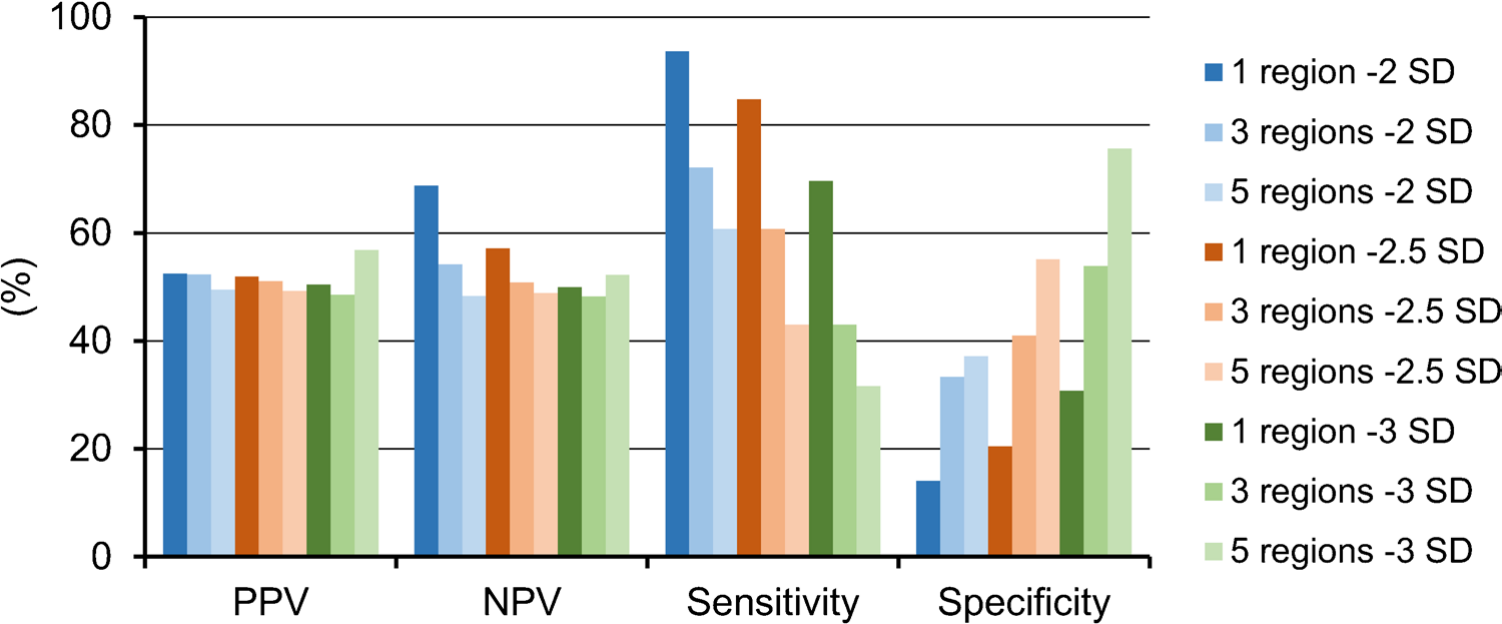
Performance of a single region hypoperfusion classifier for detection of 4RTs (*n* = 78) in contrast to other neurodegenerative disorders (*n* = 79). A single perfusion image was judged as a 4RT-like pattern when one of 21 target regions (Table 2) was below the mean value – 2 SD of healthy controls. Bootstrapping was performed with three and five regions as well as with − 2.5 and − 3.0 SD thresholds. PPV = positive predictive value; NPV = negative predictive value

We tested if the regions with significant hypoperfusion in 4RTs provide the opportunity to specifically identify individual patients with 4RTs in a real-world cohort of patients with neurodegenerative disorders referred to tau-PET at a tertiary center between 10/2018 and 4/2021. Hypoperfusion in at least one of the identified brain regions was present in 93.7% of individuals with 4RTs but also in 85.9% of patients with other neurodegenerative disorders (Fig. 2). Bootstrapping with three and five affected regions as well as with MV-2.5 SD and MV-3.0 SD thresholds resulted in improved specificity but reduced sensitivity. Overall, positive and negative predictive values for a 4RT in this cohort were low, regardless of the combination of region number and threshold (PPV: 51.4 ± 2.5 / NPV: 53.2 ± 6.5; Fig. 2).

### 4RT-related perfusion pattern expression may facilitate discrimination of patients with 4RTs additive to the 4RT-related tau pattern expression

Next, we asked if the pattern of perfusion facilitates discrimination of 4RTs from other neurodegenerative disorder more specifically than single brain regions. Thus, we performed a PCA [20] and calculated 4RT-related pattern expression scores for perfusion and tau for the [^18^F]PI-2620 scan of each subject which were subject to a subsequent ROC analysis.

**Fig. 3:**
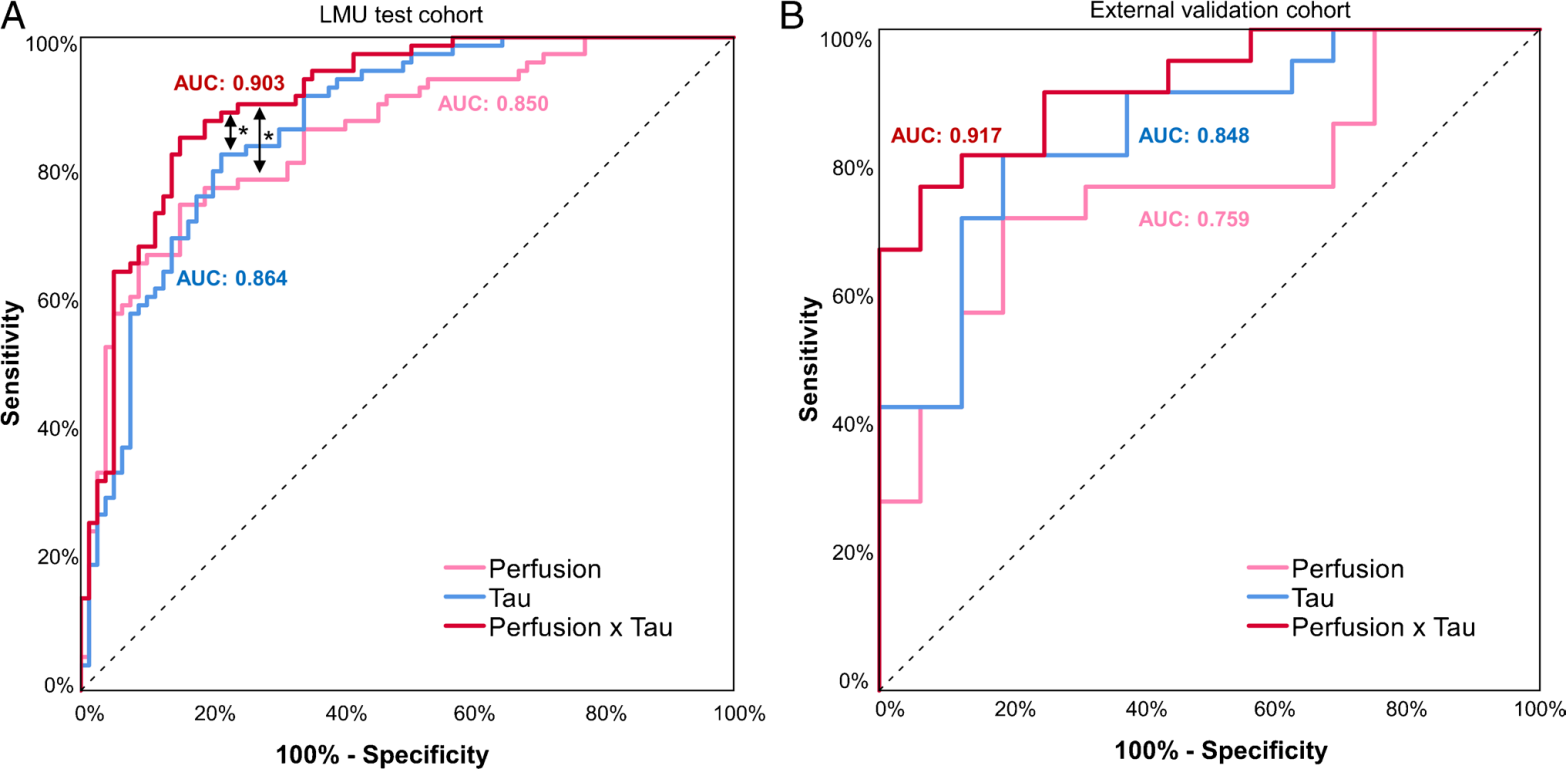
Exploratory discrimination of 4RTs and other neurodegenerative disorders by perfusion and tau pattern expression. The LMU sample consisted of *n* = 78 4RTs and *n* = 79 other neurodegenerative disorders (**A**) whereas the independent validation cohort consisted of *n* = 21 4RTs and *n* = 16 other neurodegenerative disorders (**B**). Area under the curve (AUC) values were determined using a receiver operating curve (ROC) analysis. * *p* < 0.05 for non-parametric comparison of ROC curves

The 4RT-related perfusion pattern expression showed potential for discrimination of patients with 4RTs from patients with other neurodegenerative disorders (AUC 0.850 [95%-CI: 0.790–0.910]; *p* < 0.001). Discriminatory performance of the 4RT-related tau pattern expression was similar (AUC 0.864 [95%-CI: 0.807–0.921]; *p* < 0.001) with no statistical AUC difference of the discriminatory power of perfusion and tau pattern expression (*p* = 0.702). A combined perfusion-tau expression score increased the discriminatory power of the ROC analysis to an AUC of 0.903 (95%-CI: 0.855–0.950; *p* < 0.001), which suggests a statistical additive value in contrast to stand-alone perfusion (*p* = 0.011) or tau (*p* = 0.035) pattern expression (Fig. 3A). Transfer of the trained PCs to an independent external validation dataset mirrored the findings of the main cohort, indicating an AUC of 0.917 for the combined perfusion-tau pattern expression (Fig. 3B).

### 4RT-related perfusion pattern expression correlates with cross-sectional clinical severity

**Fig. 4:**
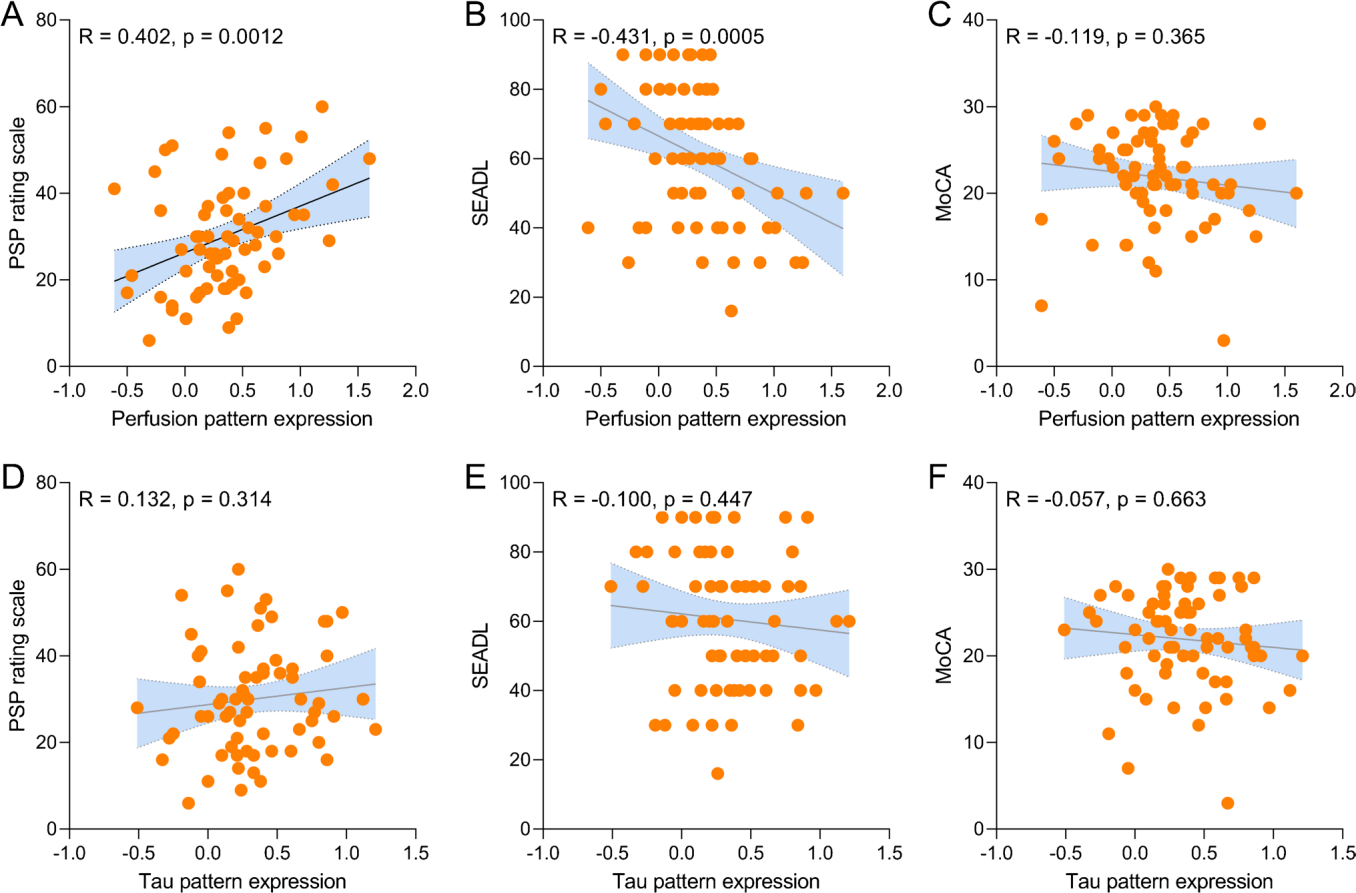
Associations of perfusion (top) and tau pattern expression (bottom) with clinical severity of 4RTs. Pearson’s correlation coefficients (*R*) derived from patients with 4RTs with available PSP rating scale data (*n* = 65, **A**, **D**), Schwab and England activities of daily living (SEADL, *n* = 66, **B**, **E**) and Montreal cognitive assessment (MoCA, *n* = 69, **D**, **F**). Correlations were controlled for age, sex, and disease duration (symptom onset)

We endeavoured to determine if perfusion alterations in 4RTs could be used as a biomarker of clinical severity. Thus, we correlated 4RT-related perfusion pattern expression with clinical scales cross-sectionally and compared the findings with the associations of 4RT-related tau pattern expression with clinical severity. 4RT-related perfusion pattern expression (i.e. perfusion deficit) was positively associated with PSP rating scale (*R* = 0.402; *p* = 0.0012) after controlling for age, sex, and disease duration (Fig. 4A). A negative association was observed between 4RT-related perfusion pattern expression and activities of daily living (*R* =  − 0.415; *p* = 0.0005; Fig. 4B), whereas no associations were observed between 4RT-related perfusion pattern expression and MoCA test performance (*R* =  − 0.119; *p* = 0.365; Fig. 4C). There were no significant associations between 4RT-related tau pattern expression and clinical severity (Fig. 4D–F). A validation analysis using only patients fulfilling the MDS PSP criteria mirrored associations between perfusion pattern expression and clinical severity (Supplemental Fig. 2).

## Discussion

In this cross-sectional study, we found that early-phase [^18^F]PI-2620 imaging yielded a valuable surrogate biomarker for perfusion alterations in 4RTs. The observed pattern of hypoperfusion in patients with 4RTs, as compared to healthy controls, matched the known topology of neuronal dysfunction in PSP and CBS. However, our study indicated that only consideration of combined brain regions has potential to facilitate discrimination of 4RT patients from patients with other neurodegenerative disorders that underwent an equal clinical workup, since single region hypoperfusion was not specific enough in 4RTs. Furthermore, we observed that combining perfusion and tau pattern information may have an additive value for the discrimination of 4RTs from other neurodegenerative disorders compared to each pattern alone. Finally, we observed stronger associations between 4RT-related perfusion pattern expression with clinical severity scales when directly compared to corresponding tau deposition. This implies that perfusion imaging could facilitate an objective read-out of disease progression of neurodegeneration in 4RTs and needs to be tested in longitudinal studies with the goal of validation as an endpoint for clinical trials.

The first goal of this study was to validate [^18^F]PI-2620 perfusion imaging for detection of regional neuronal dysfunction in 4RTs. Our previous study found a strong correlation between early static SUVr and R1 of [^18^F]PI-2620 with FDG-PET in a mixed population of neurodegenerative disorders [11]. Thus, we hypothesized that early static SUVr of [^18^F]PI-2620 facilitates the detection of known neuronal injury patterns of 4RTs against healthy controls. We selected 0.5–2.5 min SUVr since this methodology can be achieved by a simple dual-phase [^18^F]PI-2620 protocol, readily providing images for clinical interpretation without high sophisticated reconstruction and analysis methodology. In line with the known patterns of neuronal injury that were detected by perfusion imaging or FDG-PET in 4RTs [6, 21–23], we found a fronto-temporal and subcortical hypoperfusion with predominance in the thalamus, the caudate nucleus, and the anterior cingulate cortex at the group level of 4RTs against healthy controls. The putamen and the globus pallidus indicated a non-significant hyperperfusion, which was consistent with the regionally elevated time-activity-curves in the basal ganglia of patients with PSP within the perfusion phase [1]. This general pattern of perfusion in a mixed cohort of patients with 4RTs likely represents the least common denominator of perfusion alterations regardless of distinct clinical features among subgroups. Future studies should interrogate the associations between varying phenotypes of patients with 4RTs and resulting deviations from this general perfusion pattern.

On the group level, statistical analysis indicated satisfactory sensitivity of [^18^F]PI-2620 perfusion imaging for detection of 4RTs. We challenged the methodology by a mixed sample of 4RTs and other neurodegenerative diseases and used a threshold-based multiregion classifier. Here, we found only low specificity of [^18^F]PI-2620 perfusion imaging and very limited PPVs and NPVs for detection of 4RTs (average PPV/NPV < 60%). In line, low specificity of perfusion imaging and FDG-PET were consistently reported when different neurodegenerative disorders were evaluated against each other instead of comparing against healthy controls [24]. Our findings support regional similarity of hypoperfusion among diseases with partially similar clinical phenotype such as PSP-F and FTD or CBS and asymmetric AD. Thus, our findings were not surprising and emphasized the need for more detailed analyses of neuronal injury patterns [25]. Indeed, several studies successfully investigated data-driven metabolic network-based classification algorithms for discrimination of atypical Parkinsonian syndromes [25–27]. Here, sensitivity, specificity, PPV, and NPV for differential diagnosis of different parkinsonian syndromes were consistently > 80% in an automated image-derived classification procedure [25]. Importantly, one of these studies found that metabolic expression patterns did not differ between patients with PSP and patients with CBS [26] which supports pooling of 4RTs [28]. This was also justified since the majority of patients with CBS of our sample also fulfilled the MDS PSP criteria [9]. Interestingly, in our clinically pre-diagnosed cohort, the perfusion 4RT-related pattern expression showed potential for discrimination of patients with 4RTs from patients with mixed neurodegenerative diseases (AUC: 0.850). This suggests that consideration of whole brain patterns facilitates improved discrimination when compared to consideration of single regions with strongest hypoperfusion in 4RTs. This indicated the presence of disease-specific pattern apart from the regions with severe neurodegeneration and our validation cohort substantiated the usefulness of the determined networks. Furthermore, midbrain glucose hypometabolism to FDG-PET and midbrain atrophy in structural MRI were already acknowledged as supportive imaging biomarkers for diagnosis of PSP [9, 29]. In conclusion, perfusion pattern expression shows promise for 4RT discrimination in comparison to the multi-region classifier discussed above. Interestingly, a recent [^18^F]FP-CIT study similarly indicated that the early-phase of a brain PET ligand facilitates quantification of a metabolic network expression surrogate [30].

Subsequently, we tested if the combination of early and late phase 4RT pattern expressions of [^18^F]PI-2620 provide an additive value. Assuming that early-phase [^18^F]PI-2620 imaging provides the neuronal injury pattern [11] and late-phase [^18^F]PI-2620 imaging delivers information on tau aggregation [1, 31], we hypothesized a complementary gain of information. As a limitation it needs to be considered that [^18^F]PI-2620 binding in patients with 4RTs [1, 2] was not yet confirmed by autopsy in patients that underwent PET. Nevertheless, our data suggest an additive value for the combination of pattern expression in comparison to stand-alone perfusion or tau for the discrimination of 4RTs against other neurodegenerative disorders. Higher sensitivity of perfusion and higher specificity of tau pattern expression fit into the concept of “(N)” and “T” biomarker information, meanwhile well established for AD [32]. A strength of this comparison is the head-to-head evaluation of perfusion and assumed tau information in a relevant number of cases with clinically diagnosed 4RTs, according to current diagnosis criteria. As a limitation, it needs to be considered that we used 20–40-min static SUVr for assessment of tau pattern expression [15], and not the gold standard kinetic modeling approach. The focus of this research aimed to generate data that can be used in a clinical routine setting which is easier to accommodate by static windows. Therefore, it needs to be considered that the used 20–40-min static SUVr can be influenced by altered cerebral blood flow [33].

Additionally, in this study, AUC values are assessed in an already clinically diagnosed cohort with clinical evaluation being the current standard for diagnosis, which limits the value of the individual AUC values to hypothesis generating data. Therefore, here we primarily compare the additional value of combined tau and perfusion expression pattern against each pattern on their own, while prospective studies in a cohort of patients suspected to suffer from neurodegenerative disease will be needed to properly test the AUC values of tau and perfusion pattern expression against clinical diagnosis as gold standard for identification of 4RT patients. Our assessment of AUC values in an already prediagnosed cohort strongly support the hypothesis of pattern expressions being valuable biomarkers in 4RT but need to be followed up on and tested in the prospective study design.

We observed a significant association between 4RT-related perfusion pattern expression and clinical severity in our patient cohort with 4RTs. This association was specifically observed with PSP rating scale scores and activities of daily living (SEADL) but not apparent for cognitive screening (MoCA). Therefore, our findings indicate that the regional networks involved in 4RT-related perfusion pattern expression have stronger associations with gait, bulbar, limb motor, and ocular motor features than cognitive domains captured by MoCA. We note that MoCA was not developed as a dedicated screening tool for 4RTs which might limit its interpretation. Fitting to patterns of atrophy, we observed congruent decreases of early- and late-phase [^18^F]PI-2620 PET in regions near to the ventricles (i.e. caudate). Thus, it is likely that the detected lower tracer binding in these regions is not only related to hypoperfusion but also to partial volume effects, which was not entirely recoverable by PVEC. Based on our findings, we hypothesize that 4RT-related perfusion pattern expression could be a relevant biomarker for clinical progression in 4RTs which deserves testing in longitudinal studies. This could be relevant for monitoring of therapy trials since associations between different tau-related biomarkers and clinical progression in 4RTs were lower or not present in earlier cross-sectional [1, 3, 34] and longitudinal [35] studies. We note that an a priori available 4RT-related expression pattern of the [^18^F]PI-2620 perfusion phase was not available. Thus, it was necessary to use our large cohort as a training set with only a small validation set available. Longitudinal studies will aid deciphering the pathophysiology underlying the association of detected 4RT-related perfusion pattern with clinical symptoms and symptom progression. The data presented here suggests, that not tau depositions but rather resulting neuronal cell loss, i.e. perfusion, predicts symptom development and progression. Prospective investigations will be needed to understand the interplay of tau pathology, perfusion deficits, and clinical disease presentation in the 4RT disease spectrum. As a limitation, we acknowledge that autopsy confirmation of clinical diagnosis was only available in few patients. Thus, the analyses of the manuscript rely on clinical diagnosis, supporting biomarkers, and confirmation during clinical follow-up, which implies a limited number of wrong diagnoses, given by the nature of an observational study.

## Conclusions

Our data indicate that [^18^F]PI-2620 perfusion imaging is sensitive for detection of regional hypoperfusion in 4RTs. The perfusion pattern expression of 4RTs may provide an additive value to tau pattern expression for the discrimination of 4RTs from other neurodegenerative disorders and correlates closer with clinical severity (i.e. PSP rating scale) and everyday life function/activity (i.e. SEADL) when compared to tau pattern expression alone.

## Supplementary Information

Below is the link to the electronic supplementary material.

Supplementary file1 (DOCX 5959 KB)
