# Supplementary material for: Additive value of [18F]PI-2620 perfusion imaging in progressive supranuclear palsy and corticobasal syndrome
Source: Eur J Nucl Med Mol Imaging. 2022 Sep 14;50(2):423–34. doi: 10.1007/s00259-022-05964-w (PMC9816230; doi:10.1007/s00259-022-05964-w)
Supplement: Supplementary file 1 — Supplementary file1 (DOCX 5959 KB) [file 259_2022_5964_MOESM1_ESM.docx]

**Supplement**

**Supplemental Figure 1**


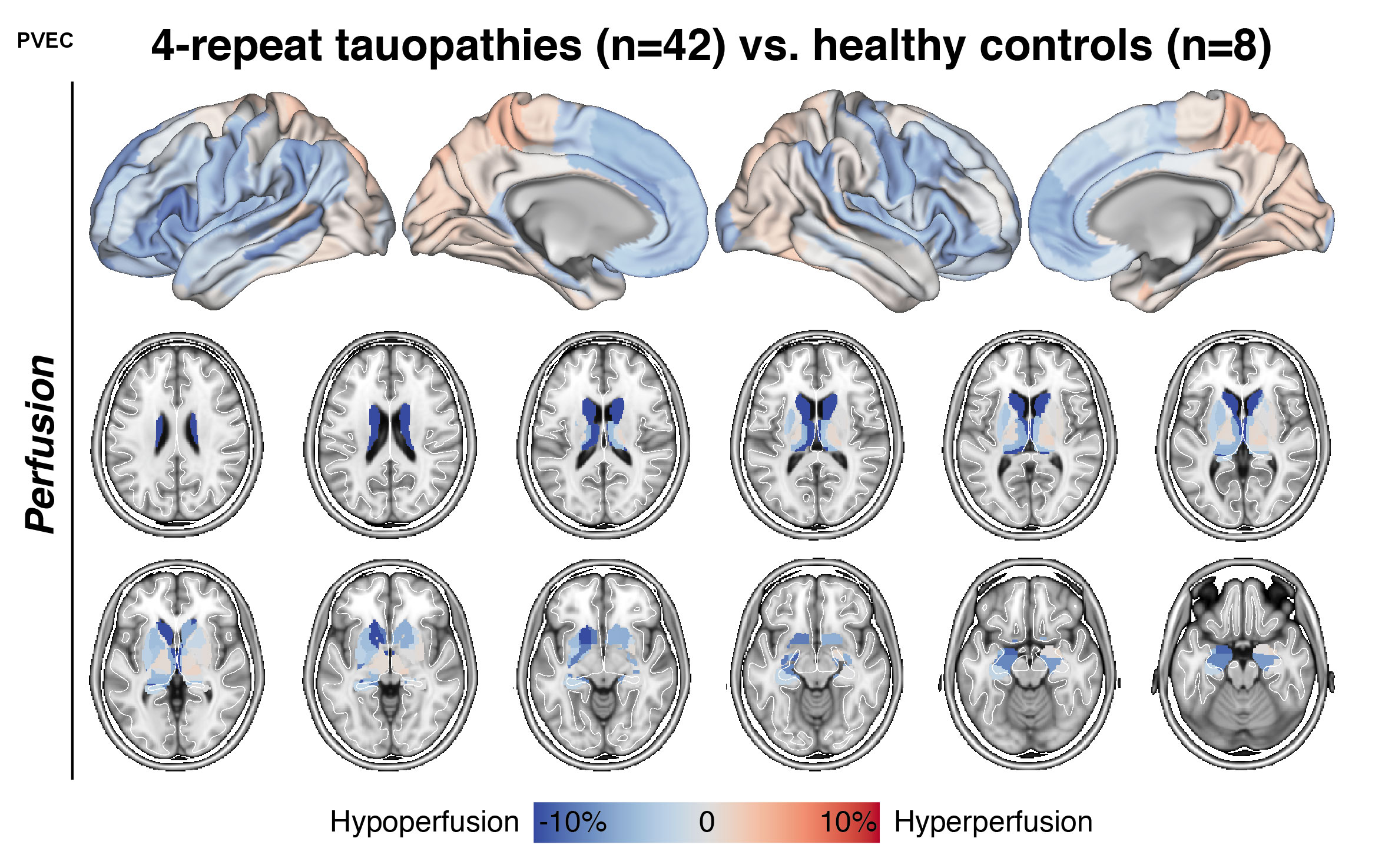

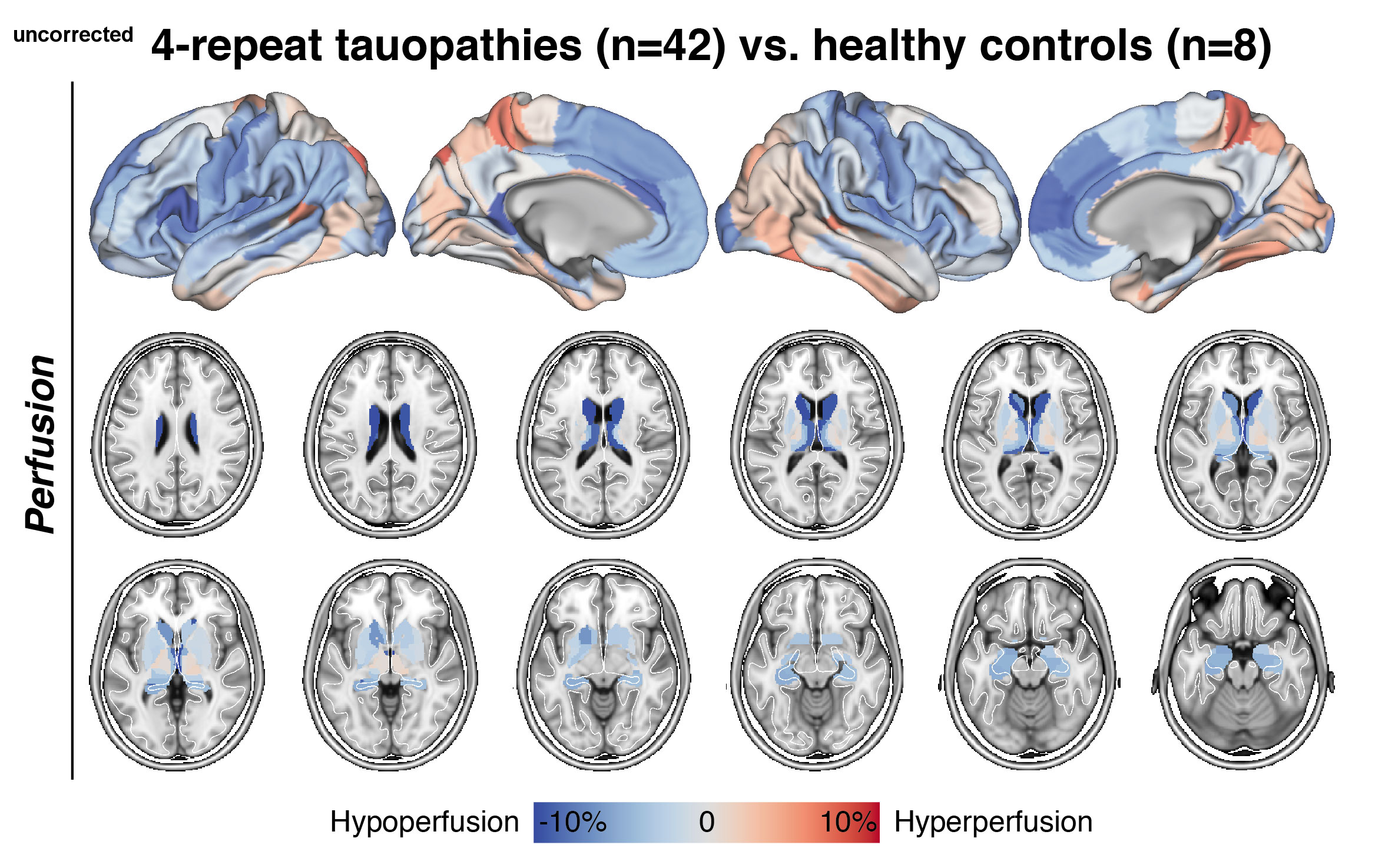


**Supplemental Figure 1 – Regional pattern of perfusion alterations in a subset of 4RTs with available T1 3D MRI (n=42) when compared to healthy controls with available T1 3D MRI (n=8) after Geometric Transfer Matrix (GTM) partial volume effect correction (PVEC, top) in comparison to uncorrected data (bottom).** Percentage differences of perfusion imaging are illustrated without significance thresholding for cortical (surface projections) and subcortical (axial slices) Brainnetome regions. Pons scaling was used to minimize PVEC effects on the reference tissue. Regional association between uncorrected and PVE corrected data was R=0.87, p<0.001.

**Supplemental Figure 2**


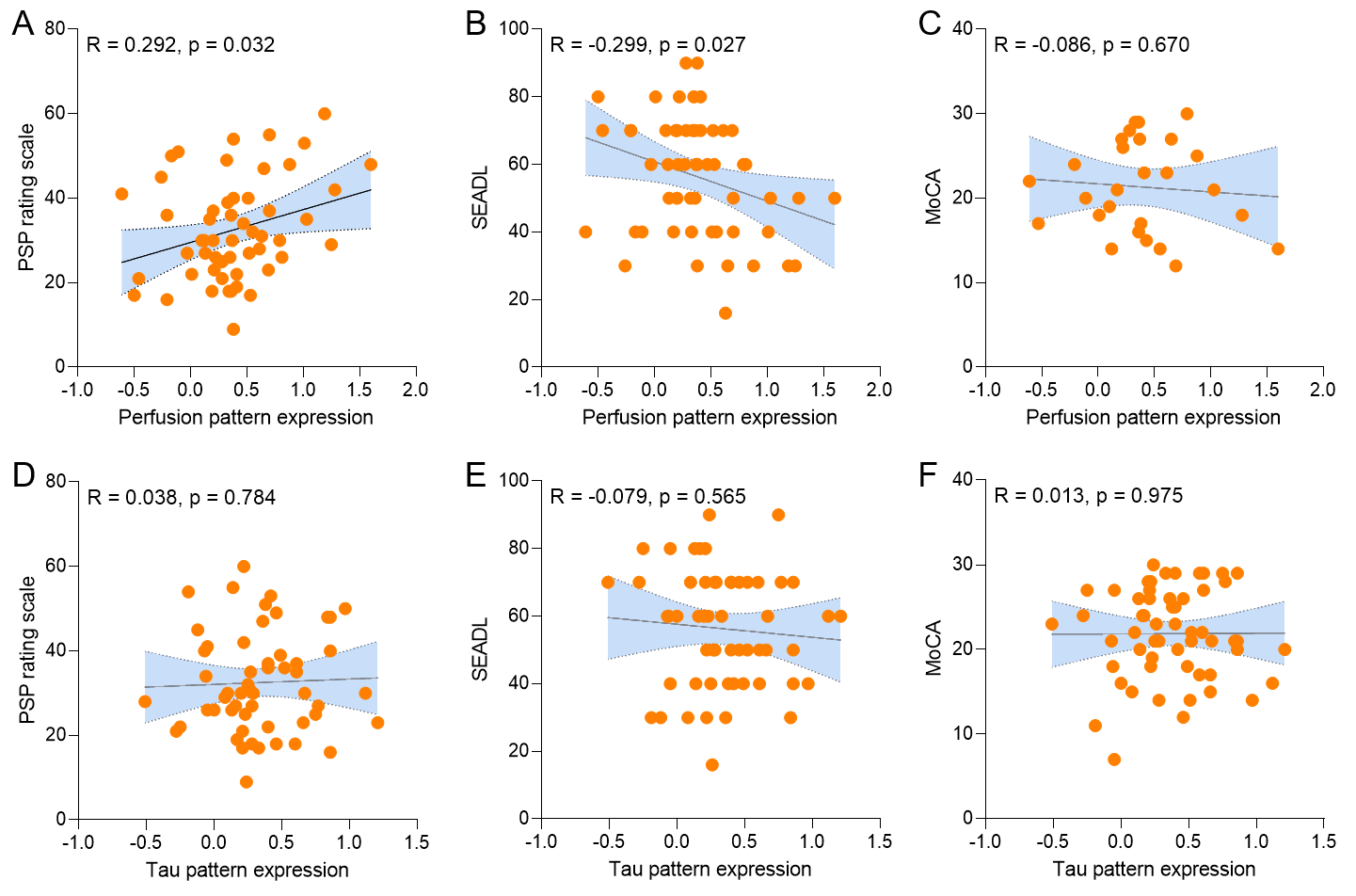


**Supplemental Figure 2 – Associations of perfusion and tau pattern expression with clinical severity of patients with PSP.** Pearson’s correlation coefficients (R) derived from patients with PSP with available PSP rating scale (n=54; **A,D**), Schwab and England activities of daily living (SEADL, n=55; **B,E**) and Montreal cognitive assessment (MoCA, n=57; **C,F**). Correlations were controlled for age, sex, and disease duration (symptom onset).
